# Anti‐Amyloid Immunotherapy for AD: A Potential Link between ARIA and Complement

**DOI:** 10.1002/alz.093183

**Published:** 2025-01-03

**Authors:** Praveen Bathini, Stephan Schilling, Cynthia A. Lemere

**Affiliations:** ^1^ Brigham and Women’s Hospital; Harvard Medical School, Boston, MA USA; ^2^ Fraunhofer Institute for Cell Therapy and Immunology, Halle Germany

## Abstract

**Background:**

Anti‐amyloid antibodies have been associated with amyloid‐related‐imaging‐abnormalities (ARIA) in AD patients, causing vasogenic edema and microhemorrhages, especially in ApoE4 carriers. Here, we compared recombinant 3D6‐L, a murine version of bapineuzumab, and an isotype control IgG2a monoclonal antibody (mAb) to investigate potential mechanisms, including complement activation, involved in these side effects (ARIA‐H or microhemorrhages) following passive immunization.

**Method:**

Plaque‐rich 16.5‐mo‐old APP/PS1dE9; hApoE4 mice were treated weekly (i.p.) for 13 weeks with ∼15 mg/kg (500 µg) 3D6‐L or IgG2a (n = 5 per group). amyloid‐beta (Aβ), ApoE, and complement C3 levels from the brain homogenates were determined using ELISA and microhemorrhages with Prussian‐blue staining. Additionally, 20‐month‐old APP/PS1dE9;hApoE4 mice underwent a shorter 7‐week passive immunization (500 µg/week 3D6‐L or IgG2a, n = 3 per group) followed by RNAseq to evaluate differentially expressed genes (DEGs) in the brain and FACS sorted CD31^+^ vascular endothelial cells. Pathological analyses are underway.

**Result:**

In the 13‐week study, 3D6‐L significantly reduced guanidium‐HCL‐soluble Aβx‐42 and Aβx‐40; ApoE levels showed a trend for reduction. Soluble C3 levels rose significantly, correlating with increased microhemorrhages in leptomeninges and large blood vessels in the cortex and cerebellum. In the shorter immunization study, brain transcriptome revealed significant upregulation of many genes related to complement activation (*C1qa, C1qb, Cq1qc, C4b, C3*), glial activation (*GFAP*), IgG receptor activity (*Fcgr1, Fcgr2, Fcgr3*), lysozyme (*Lyz2*) and Galectin‐3 (*LGALS3*). Endothelial cell transcriptome revealed increased expression of genes involved in inflammation and lipid metabolism. Some of these include *IL1r1, Cxcl12, Lcn2, Abca1, CH25H, vWf*, and *Cfh*. Notably, 3D6‐L induced microhemorrhages, microbleeds, and our preliminary analysis indicate an increase in cerebral amyloid angiopathy (CAA)‐associated complement activation and endothelial inflammation. Based on our RNAseq data, we are investigating potential markers including, iC3b, C5b‐9, MMP‐9, and vWf involved in immunotherapy‐associated microhemorrhages.

**Conclusion:**

Passive immunization against Aβ using 3D6‐L amplified CAA‐associated complement activation and altered expression of genes related to extracellular matrix degradation and vascular inflammation. The observed microhemorrhages appear to result from complement‐mediated vascular inflammation induced by the binding of 3D6‐L to vascular amyloid. These findings suggest potential biomarkers or targets for therapeutic interventions in the context of anti‐Aβ immunization.

